# Resistance mutations that distinguish HIV-1 envelopes with discordant VRC01 phenotypes from multi-lineage infections in the HVTN703/HPTN081 trial: implications for cross-resistance

**DOI:** 10.1128/jvi.01730-24

**Published:** 2025-01-16

**Authors:** Paula Cohen, Bronwen E. Lambson, Nonhlanhla N. Mkhize, Chivonne Moodley, Anna E. J. Yssel, Thandeka Moyo-Gwete, Talita York, Asanda Gwashu-Nyangiwe, Nonkululeko Ndabambi, Ruwayhida Thebus, Michal Juraska, Allan C. deCamp, Brian D. Williamson, Craig A. Magaret, Peter B. Gilbert, Dylan Westfall, Wenjie Deng, James I. Mullins, Lynn Morris, Carolyn Williamson, Penny L. Moore

**Affiliations:** 1 SA MRC Antibody Immunity Research Unit, School of Pathology, Faculty of Health Sciences, University of the Witwatersrand, Parktown 37708 , Johannesburg, South Africa; 2 Centre for HIV and STIs, National Institute for Communicable Diseases (NICD), a division of the National Health Laboratory Service 70687 , Johannesburg, South Africa; 3 Division of Medical Virology and Institute of Infection Disease and Molecular Medicine, Department of Pathology, Faculty of Health Sciences, University of Cape Town 37716 , Cape Town, South Africa; 4 Vaccine and Infectious Disease Division and Statistical Center for HIV/AIDS Research and Prevention, Fred Hutchinson Cancer Center 7286 , Seattle, Washington, USA; 5 Department of Biostatistics, University of Washington 312753 , Seattle, Washington, USA; 6 Biostatistics Division, Kaiser Permanente Washington Health Research Institute 343041 , Seattle, Washington, USA; 7 Department of Medicine, University of Washington 205280 , Seattle, Washington, USA; 8 Department of Global Health, University of Washington 150331 , Seattle, Washington, USA; 9 Centre for the AIDS Programme of Research in South Africa (CAPRISA), University of KwaZulu-Natal 56394 , Durban, South Africa; 10 National Health Laboratory Service 70685 , Cape Town, South Africa; University Hospital Tübingen, Tübingen, Germany

**Keywords:** HIV-1, broadly neutralizing antibodies, VRC01, escape, acquisition, bottleneck

## Abstract

The Antibody Mediated Prevention (AMP) trials showed that passively infused VRC01, a broadly neutralizing antibody (bNAb) targeting the CD4 binding site (CD4bs) on the HIV-1 envelope protein (Env), protected against neutralization-sensitive viruses. We identified six individuals from the VRC01 treatment arm with multi-lineage breakthrough HIV-1 infections from HVTN703, where one variant was sensitive to VRC01 (IC_50_ < 25 ug/mL) but another was resistant. By comparing Env sequences of resistant and sensitive clones from each participant, we identified sites predicted to affect VRC01 neutralization and assessed the effect of their reversion in the VRC01-resistant clone on neutralization sensitivity. In four pairs, a single mutation restored partial or full sensitivity to VRC01, whereas in the fifth participant, transfer of the entire β23-V5 loop was required. No VRC01 resistance mutations could be identified in the sixth participant, with the discordant clones differing by >100 amino acids. Mutations responsible for the differential neutralization phenotypes occurred at distinct sites across Env, including residues in loop D, the CD4-binding loop, and between the β23 and V5 loops. Analysis of deep sequencing *env* data showed that VRC01 resistance was likely the property of the acquired virus, rather than occurring through post-acquisition evolution. Although VRC01-resistant parental clones generally retained sensitivity to other CD4-binding site bNAbs, they were less potently neutralized than the VRC01-sensitive clones. In conclusion, VRC01 resistance mutations occurred through multiple mutational pathways, but sensitivity to second-generation CD4bs bNAbs was retained even in VRC01-resistant transmitted viruses, confirming the potential of these bNAbs for HIV-1 prevention studies.

**IMPORTANCE:**

The Antibody Mediated Prevention (AMP) trials provided proof of principle that VRC01, a CD4-binding site (CD4bs) HIV-1 broadly neutralizing antibody (bNAb), prevented the acquisition of antibody-sensitive viruses. However, understanding common mutations that confer resistance to different bNAbs provides important insights into the genetic barrier to resistance. Here we studied six AMP trial participants with breakthrough infections mediated by multiple viral lineages with discordant VRC01 sensitivity. We identified different mutations across the CD4-binding site that conferred resistance to VRC01 and showed that these mutations were a property of the acquired virus, rather than a result of post-acquisition evolution. We found that although VRC01 resistance was associated with reduced neutralization potency of second-generation CD4-binding site bNAbs, overall neutralization sensitivity was generally retained, which is promising for future use of such bNAbs in clinical trials.

## INTRODUCTION

The HIV-1 pandemic continues to be a major public health threat, and a preventive HIV-1 vaccine has yet to be developed. Several vaccine approaches seek to elicit broadly neutralizing antibodies (bNAbs) which develop in some individuals living with chronic HIV, and target highly conserved viral epitopes (1). In the absence of a vaccine that triggers such antibodies, the HIV-1 prevention field has explored the use of passively administered bNAbs for HIV-1 prevention (2). While many bNAbs have high levels of coverage against circulating viruses, viral resistance represents a challenge, especially given the high levels of diversity within the HIV-1 envelope protein (Env) (3). Understanding the mechanisms that confer viral resistance to bNAbs is important to both passive immunization studies and vaccine design.

Proof of the principle that bNAbs could prevent HIV-1 acquisition has been shown in several studies in non-human primate models, which led to two large clinical trials to assess this concept (4–6). The Antibody Mediated Prevention (AMP) (HVTN703/HPTN081 and HVTN704/HPTN085) studies evaluated the effectiveness of two dose levels (10 mg/kg and 30 mg/kg) of the CD4-binding site-directed bNAb, VRC01, in preventing HIV-1 acquisition (4). Although the AMP studies did not show overall prevention efficacy, a pooled analysis of both dose groups across both trials showed that VRC01 was 75% efficacious against viruses that were highly sensitive to VRC01 (IC_80_ <1 ug/mL). The AMP studies thus provided the first proof of concept that bNAbs could prevent HIV-1 infection but also provided a unique opportunity to define viral features in breakthrough infections that conferred resistance to VRC01.

VRC01 is a class-defining bNAb targeting the highly conserved CD4-binding site, which is required for viral binding to its host cell receptor (7). VRC01 has considerable cross-clade neutralizing breadth, neutralizing 65% to 81% of virus strains in contemporaneous subtype B and C panels at IC_80_ <10 ug/mL, but only 30% of viruses were found to be highly sensitive to neutralization at IC_80_ <1 ug/mL (4, 7). Structural studies have revealed that VRC01-class bNAbs partially mimic CD4 in their interaction with gp120 (7–9). VRC01 contact residues are found in loop D, the CD4-binding loop, and the β23-V5 loop regions of gp120, and mutations at these sites are associated with resistance (9–11). A meta-analysis of 611 HIV-1 gp160 pseudoviruses from the CATNAP database (https://www.hiv.lanl.gov) identified 24 HIV-1 Env sequence features as predictive of VRC01 neutralization resistance (12 residues, five potential N-linked glycosylation site motifs, and seven viral features including length of gp120 or variable loops, number of glycans, and number of cysteines) (12). Some of these sites have been identified in chronically infected individuals, who, when treated with VRC01, transiently controlled viral replication, and in whom rebound was associated with the emergence of resistance mutations (13, 14). Previous studies also showed that escape from CD4-binding site antibodies including VRC01 may confer a fitness cost but that compensatory mutations can emerge to accommodate resistance (14, 15).

VRC01 is a first-generation bNAb recognizing the CD4-binding site (CD4bs) but several broader and more potent CD4bs antibodies were subsequently isolated and/or engineered (16–18). The ability of VRC01 resistance mutations to confer resistance to other VRC01-class CD4bs antibodies is variable. Lynch et al showed that VRC01 resistance conferred cross-resistance to VRC-PG04, VRC-CH31, 3BNC117, 12A12, and VRC-PG20, with slight variations attributed to small epitope differences between antibodies (14). Similarly, the culturing of viruses in the presence of VRC01 led to the emergence of resistance to most other bNAbs in the same class, some of which were completely resistant to every well-characterized VRC01-class bNAb, including VRC01, NIH45-46, 3BNC117, VRC07, N6, VRC-CH31, and VRC-PG04 (19).

HIV-1 acquisitions have typically been associated with a stringent bottleneck, where it is estimated that 75% of infections were reported to be caused by outgrowth from a single viral particle (20–22). This stringent bottleneck is likely an advantage for HIV-1 vaccines, as vaccine-elicited antibodies would have to contend with limited viral variability (3, 23). The definition of a stringent bottleneck came from studies that were performed through single-genome sequencing of limited depth (~20 to 30 sequences per donor) (20, 22). Using deep sequencing, multi-lineage infections were observed in 34% of AMP participants in an analysis of a subset of the 74 primary endpoints who had sequencing data available (mean >100 sequences per participant) from the first RNA-positive visit (23), with some of these lineages having varying sensitivity to VRC01 (23). Defining the mutations that distinguish Envs with discordant VRC01 sensitivity phenotypes and their relevance for cross-resistance to other clinically important bNAbs will inform both passive and active immunization approaches.

Here we identified six participants from the HVTN 703/HPTN 081 study with multi-lineage HIV-1 breakthrough infections from the AMP study, where the isolated viruses had discordant sensitivity to the VRC01 bNAb. We identified the mutations responsible for the discordant VRC01 phenotypes and assessed the sensitivity of VRC01-resistant viruses to other clinically relevant CD4-binding site antibodies. Our data highlight that multi-lineage infections can be caused by viruses with differential sensitivity to bNAbs and that the mechanism of bNAb resistance is variable, with escape mutations occurring at multiple different sites. However, sensitivity to next-generation CD4-binding site monoclonal antibodies (mAbs) is generally well-retained even for VRC01-resistant viruses, suggesting that second-generation CD4-binding site bNAbs have clinical potential for HIV-1 prevention.

## MATERIALS AND METHODS

### Study participants from HVTN703/HPTN081 AMP trial

A total of 1,924 women in Southern Africa with an increased likelihood of HIV acquisition were enrolled in the HVTN 703/HPTN 081 AMP trial. These women were randomly allocated to receive either a placebo or one of two different doses of VRC01 infusions (low dose of 10 mg/kg and high dose of 30 mg/kg) every 8 weeks for 72 weeks (total of 10 infusions). Plasma RNA testing was done every 4 weeks to assess HIV-1 acquisition (4).

### Sequencing and lineage assignment

HIV-1 Envs were sequenced from 91 participants who acquired HIV in the HVTN703/HPTN081 AMP trial (placebo = 33, VRC01 low dose = 36, VRC01 high dose = 22), with 89 participants who acquired a clade C virus, one clade G (from Kenya) and one an A/C recombinant (South Africa) (24). Viruses were deep sequenced by PacBio Single-Molecule-Real Time sequencing as described (25). Lineages were assigned using the Phylobook tool (26). This tool generates a maximum-likelihood phylogenetic tree and displays sequence changes from the dominant sequence using a highlighter plot, with sequence similarity visualized using a matcher plot (https://phylobook.cloud/). Participants were defined as having multi-lineage infections based on phylogenetic clustering on the maximum-likelihood trees and high diversity between sequences.

### Mutagenesis and construction of envelope chimeras

Site-directed mutagenesis was performed using the QuikChange Lightning Multi Site-directed Mutagenesis Kit (Agilent Technologies, catalog #210519), as per the manufacturer’s instructions. Where multiple mutations needed to be introduced simultaneously, chimeras were constructed by inserting the β23-V5 loop of one plasmid into the Env of another, using the NEBuilder HiFi DNA assembly Cloning Kit (catalog #E5520S), as per the manufacturer’s instructions. These changes were confirmed by Sanger sequencing.

### Pseudovirus production and neutralization assays

Pseudoviruses were made by co-transfecting 293T/17 cells with 4 ug of either parental wild type or mutant Env plasmids and 4 ug of a backbone plasmid DNA (pSG3Δenv), an Env-deficient HIV-1 backbone vector (27, 28). Pseudoviral titers were measured by TCID50 assay to determine viral input for the TZM-bl neutralization assay. The viruses were tested against a panel of CD4-binding site-directed bNAbs (VRC01, N6, VRC07-523 LS, 3BNC117, 1-18, HJ16) and the amount of neutralization was based on the reduction of luciferase reporter gene expression after a single round of infection of the TZM-bl cells with the pseudoviruses. Titers were expressed as IC_50_ values, the 50% maximal inhibitory concentration, with a starting concentration of 25 ug/mL of bNAb (27, 28).

### Phylogenetic tree

A phylogenetic tree of the pseudoviruses was drawn using IQ-TREE (29) using the General Time Reversible (GTR) substitution model, with eight categories of rate heterogeneity. Branch support was approximated by ultrafast bootstrapping (30) with 1,000 replicates. The tree was rooted on V703_0865 which is a HVTN703/HPTN 081 subtype G sequence. The tree visualization was done using iTOL (31). DNA distances were calculated using Phylogenetic estimation using Maximum Likelihood (https://github.com/stephaneguindon/phyml) with GTR.

## RESULTS

### Neutralization profiles of multi-lineage infections from the HVTN703/HPTN081 AMP trial

**Fig 1:**
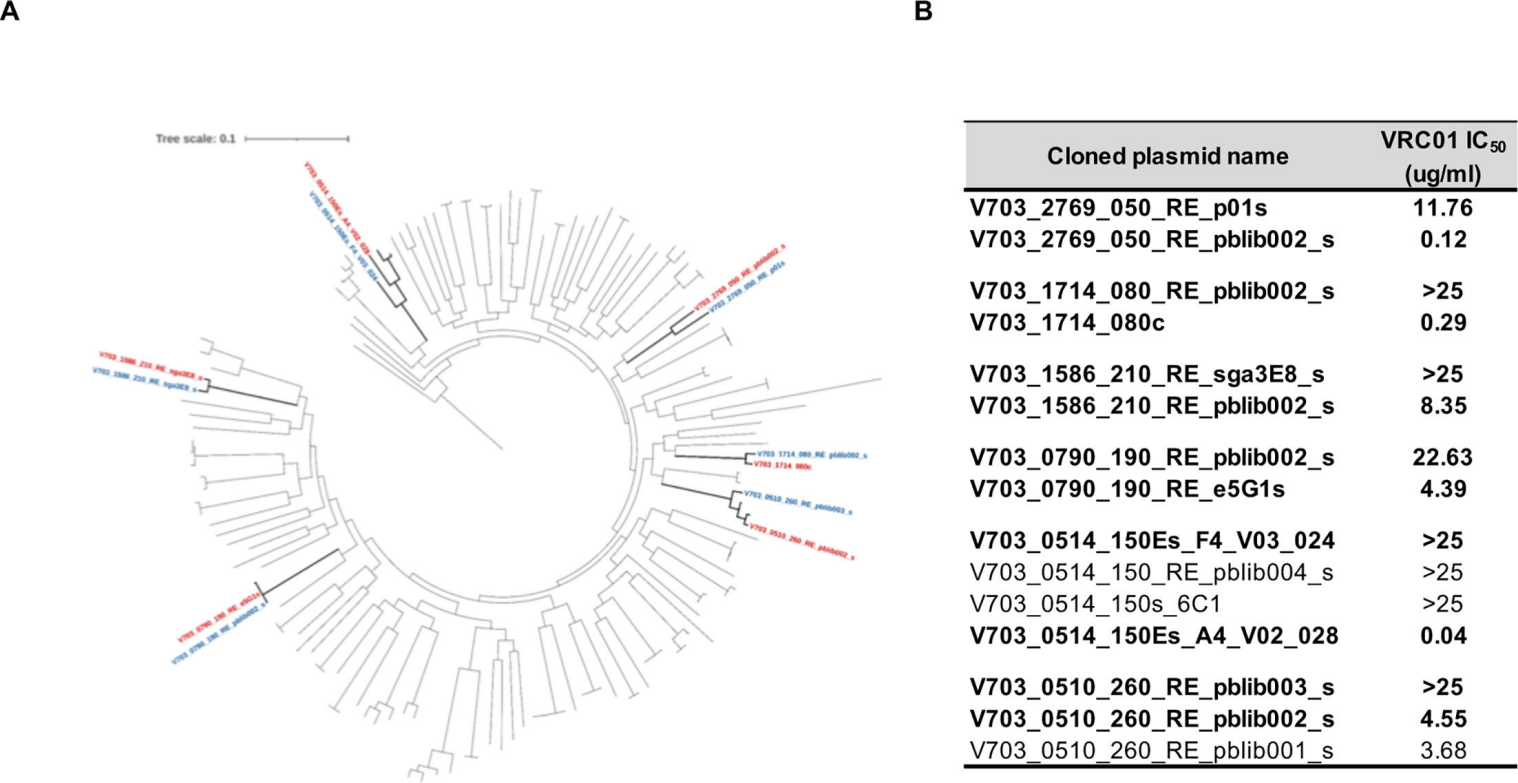
(**A**) Maximum-likelihood phylogenetic tree of the env sequences from viruses cloned from each of the HVTN703/HPTN081 AMP trial participants at the first HIV-1 RNA-positive timepoint, highlighting the Envs which represent the dominant viruses sampled (24). The six clusters highlighted in bold branches show multiple viruses isolated from the same individual, with sequences of selected pairs of VRC01-sensitive clones in red, and VRC01-resistant clones in blue. The tree scale indicates the estimated number of substitutions per site. (**B**) Neutralization sensitivity of all clones within the phylogenetic clusters, with clones selected for further study shown in bold. IC_50_ values measured in ug/mL. Discordance was defined as a greater than threefold difference in VRC01 resistance by IC_50_.

During the AMP trials, individuals were screened for HIV-1 acquisition monthly, and Envs were synthesized from viruses present at the first HIV-1 RNA-positive visit. We have previously described the VRC01 neutralization sensitivity profiles of the acquired isolated virus(es) observed in the HVTN 703/HVTN 081 trial which enrolled women in sub-Saharan Africa (23, 24). Here, we focused on six participants from the VRC01 treatment arm of this trial with multiple virus isolates that differed in their VRC01 sensitivity as measured by IC_50_ (23, 24). Figure 1A shows a phylogenetic tree of all the Env clones synthesized and tested for VRC01 sensitivity in the HVTN703/HPTN081 AMP trial. Nodes in bold indicate sequenced from the six participants in this study, with labels colored to indicate VRC01 sensitivity (red) or resistance (blue).

Figure 1B shows the VRC01 IC_50_ sensitivity of each clone (with IC_80_ shown in Fig. S1) within the six participants. For example, for participant 1714, two early lineages were identified, one of which was sensitive to VRC01 (with an IC_50_ of 0.29 ug/mL) and the other resistant (IC_50_ >25 ug/mL). Similarly for participant 0510, three VRC01-sensitive Env (with IC_50_ titers ranging from 3.68 to 4.55 ug/mL) and a single VRC01-resistant Env were identified. From each cluster, we selected a single resistant and a single sensitive clone (shown in bold, Fig. 1B and labeled in color in Fig. 1A) for further study.

### Defining VRC01-resistant mutations that distinguish HIV-1 envelopes with discordant VRC01 neutralization phenotypes

We next sought to define the mutations that distinguished the two HIV-1 Envs with discordant VRC01 neutralization phenotypes. We compared the sequences of the discordant clones (Fig. S2A through F), focusing on features that are known to impact VRC01 sensitivity, including mutations within the CD4-binding site; the length of the β23-V5 loop; and the number and position of potential N-linked glycosylation sites (12, 32). We also assessed potential sites of interest using a machine-learning-based model that we have previously shown can predict VRC01 sensitivity (as opposed to resistance) (12, 23). We identified between 1 and 7 amino acids per discordant pair in the CD4-binding site, which differed between the VRC01-discordant clones.

**Fig 2:**
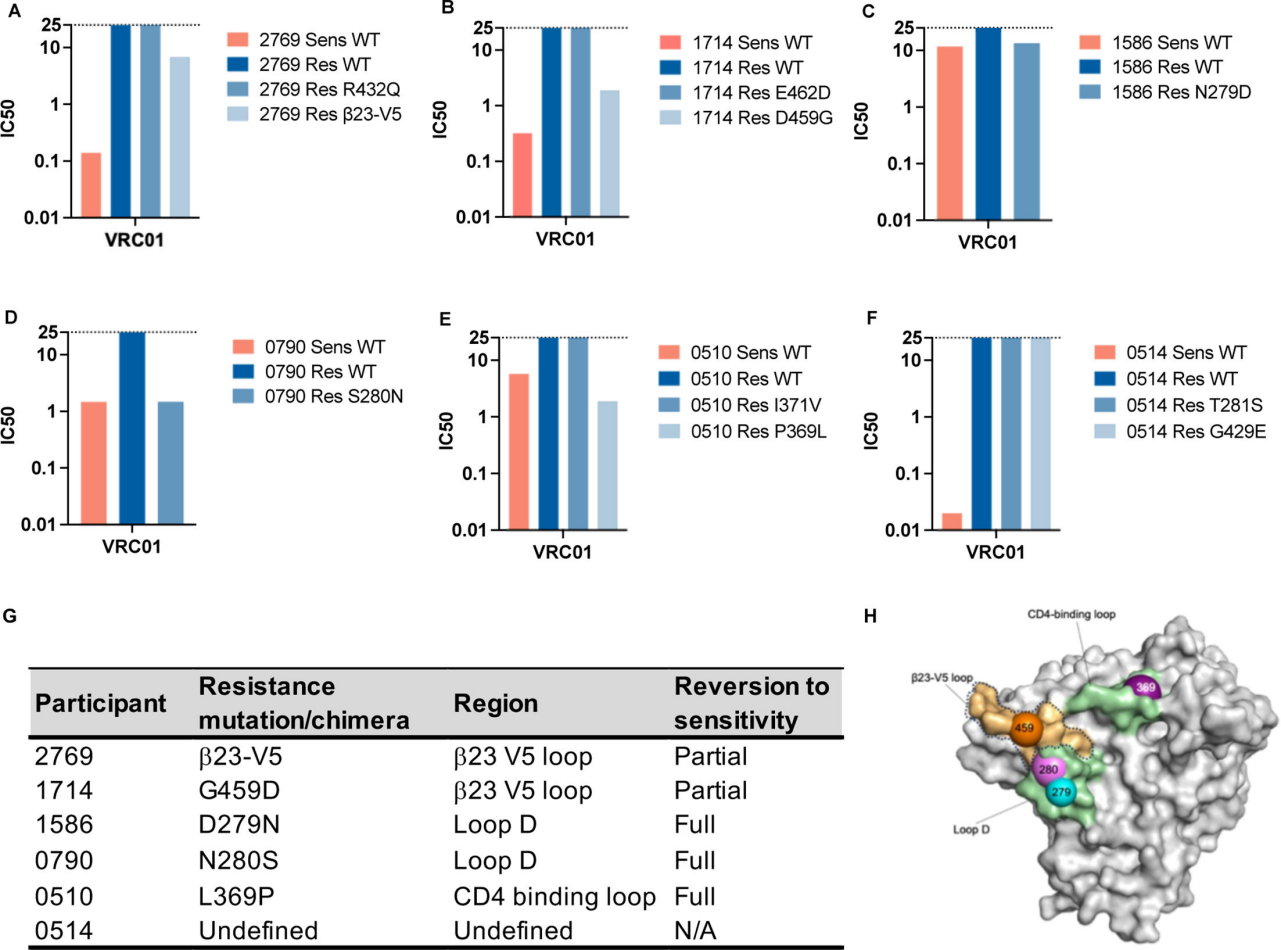
Mapping of mutations that confer resistance to VRC01. (**A–F**) The VRC01 titers of mutant pseudoviruses (shades of light blue) compared to sensitive WT (Sens WT, red) and resistant WT (Res WT, dark blue), where IC_50_ is measured in ug/mL with a max IC_50_ of >25 ug/mL (defined as resistant) marked with the dotted line. (**G**) Summary of the region and specific mutation or chimera identified for each participant associated with VRC01 resistance. Mutations that had no impact are not shown. (**H**) Visualization of the CD4-binding site, highlighted in color, within which regions/mutations associated with VRC01 resistance are highlighted. Loop D and the CD4-binding loop are colored green with the residues at sites 279 (blue), 280 (pink), and 369 (purple) highlighted, respectively, and the β23-V5 loop is colored orange with the chimera outlined by the dotted line and the 459 residues highlighted in orange. Image created in Pymol (Version 2.5.2Schrödinger, LLC) using PDB ID: 4LST.

To assess which mutations were associated with VRC01 resistance, we used site-directed mutagenesis to revert mutations in the parental (wild type [WT]) VRC01-resistant clone (Res WT) to that amino acid(s) in the parental wild-type sensitive clone (Sens WT). In the cases where more than two mutations distinguished the sequences in regions of interest, we created chimeras, transferring multiple mutations simultaneously between the discordant clones. All mutants and chimeras were sequence verified, and the TZM-bl pseudovirus neutralization assays were then performed to compare the VRC01 sensitivity of the mutated envelopes with both parental clones (Sens WT and Res WT) (Fig. 2A through F).

In two participants, 2769 (Fig. 2A) and 1714 (Fig. 2B), we identified mutations that conferred partial VRC01 sensitivity when introduced into the Res WT clones, however, these mutations were not sufficient to completely restore VRC01 sensitivity. For participant 2769, the R432Q mutation in the CD4 contact site (β20/β21) did not impact neutralization but the simultaneous transfer of the entire β23-V5 loop of the 2769 Sens WT clone into the Res WT clone (a total of three amino acid substitutions and a four amino acid deletion) resulted in a change in VRC01 titer from >25 ug/mL to 6.82 ug/mL (compared to 0.14 ug/mL for 2769 Sens WT). For 1714, the introduction of E462D mutation in the V5 loop of the WT Res clone failed to confer VRC01 sensitivity but a single D459G mutation (between the β23 and V5 loop) resulted in increased VRC01 sensitivity from >25 ug/mL (Res WT) to 1.89 ug/mL (compared to 0.32 ug/mL for 1714 Sens WT).

For three participants, 1586 (Fig. 2C), 0790 (Fig. 2D) and 0510 (Fig. 2E), we identified a single mutation that conferred full VRC01 sensitivity. In participant 1586, the introduction of an N279D mutation (in Loop D) into 1586 Res WT resulted in a similar moderately sensitive VRC01 profile to the 1586 Sens WT (13.40 ug/mL and 11.76 ug/mL, respectively). For 0790, the sensitive and resistant viruses differed only by the S280N mutation (in loop D), and when introduced into 0790 Res WT fully restored VRC01 sensitivity. In participant 0510, the introduction of a P369L mutation into 0510 Res WT resulted in a VRC01 IC_50_ of 1.89 ug/mL, which was slightly more potent than 0510 Sens WT (5.73 ug/mL), whereas an introduction of an I371V mutation had no impact on VRC01 sensitivity.

For the final participant, 0514 (Fig. 2F), neither of the single amino acid substitutions tested (T281S or G429E) impacted VRC01 sensitivity. We also constructed and tested two chimeras that transferred the CD4-binding loop or β23-V5 loop, however, neither generated functional pseudoviruses. As there were over 100 base pair differences between the two parental sequences (Supplementary 2F), we were unable to identify the mutations conferring VRC01 resistance in this participant.

In summary, in four of the six pairs of clones from multi-lineage infections, a single mutation was sufficient to revert-resistant clones to a fully VRC01 sensitive (1586, 0790, and 0510) or partially sensitive (1714) phenotype, with multiple mutations required in the case of 2769. In all five cases where functional shifts were observed, the mutations responsible for the differential neutralization phenotypes occurred at different amino acid sites within Env, such as 279 and 280 in loop D, 369 in the CD4-binding loop, and 459 between the β23 and V5 loop (Fig. 2G and H). This highlights the diverse VRC01 escape pathways that exist.

### VRC01 resistance is the property of the acquired virus, rather than post-acquisition evolution

**Fig 3:**
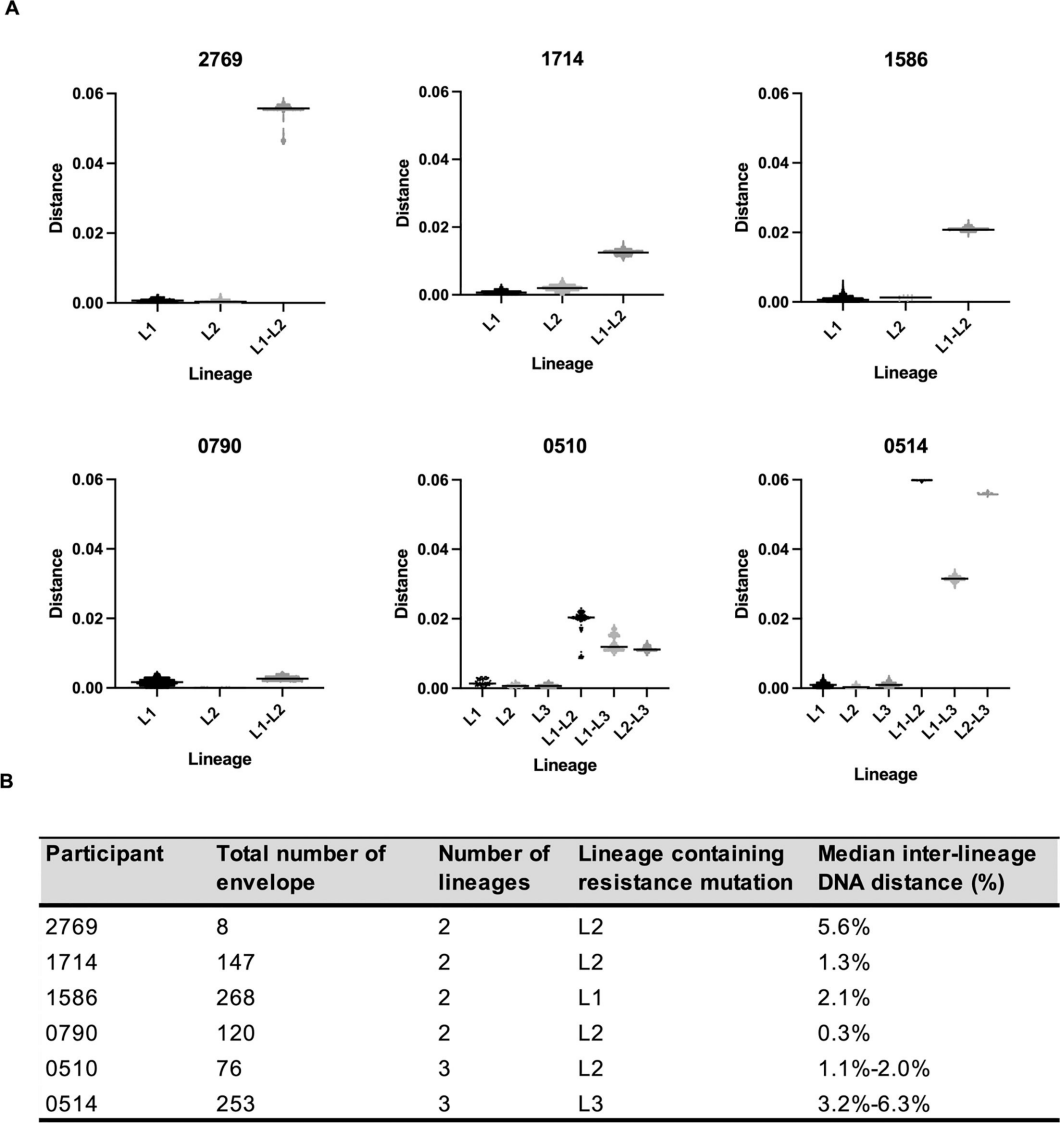
Maximum-likelihood pairwise DNA distances within and between lineages. (**A**) Pairwise DNA distance plots illustrating intra-lineage (L1, L2, or L3) and inter-lineage (**L1–L2, L1–L3, L2–L3**) DNA distances. The medians are indicated by black lines. (**B**) The number of envelope gene sequences analyzed, the number of lineages identified, the lineage containing the resistance mutation defined in panel A above, and the median distances between the lineages as a percentage, are shown.

We next sought to establish whether VRC01-resistant clones were most likely to have been transmitted, or if resistance evolved post-acquisition. Env sequences from the first point of diagnosis, generated through deep sequencing, were classified into different lineages based on their phylogenetic relatedness as well as DNA distance. Figure 3 shows the number of sequences generated from each of the six participants, the number of lineages identified, and the DNA distances within and between the lineages. Participants were shown to have acquired 2 to 3 lineages (L1, L2, and L3), and all intra-person lineages were highly homogeneous with a low DNA distance (median DNA distance <0.2%). In five out of six individuals, the viral lineages were distinct from each other (1.1% to 6.3% difference), with no overlap between the inter- and intra-lineage DNA distances. In one participant (0790), the two lineage sequences differed by only five unique distinguishing mutations, however, there was limited change within lineages (intra-lineage distance 0.1% and 0% for lineage 1 and 2, respectively) (Fig. 3). In this individual, the resistance was located on the derived lineage 2 founder virus sequence, suggesting that the resistant mutation was already present in the donor. Since the resistant and sensitive clones from all six individuals were grouped into different lineages, this suggests the resistant phenotype was the property of the acquired virus. However, we cannot exclude the possibility that resistance in 0790 emerged post-acquisition.

### Resistance to VRC01 does not confer resistance to other CD4-binding site bNAbs but is associated with reduced potency

We next investigated whether the VRC01 resistance mutations impacted neutralization sensitivity to a panel of five CD4-binding site bNAbs, three of which are VRC01-class antibodies (N6, VRC07-523LS, and 3BNC117), and two of which have different germline gene usage (1-18 and HJ16). The epitope, class, germline genetic features, origin, potency, breadth, and 276 glycan interaction of each of these mAbs are summarized in Table 1. We compared the sensitivity profiles of the parental sensitive wild type (Sens WT), parental resistant WT (Res WT), and the resistant WT clone that had been reverted to VRC01 sensitivity (Rev^Sens^).

**TABLE 1:** CD4 binding site bNAb panel^*a*^

mAb	Epitope	VRC01-class	Heavy chain gene	Light chain gene	Origin	Virus panel	% Breadth (<50 μg/mL)	Geomean potency (μg/mL)	276 glycan interaction
VRC01	CD4bs	Y	IGVH1-2	IGKV1-33	Human	993	85	0.4	N
N6	CD4bs	Y	IGVH1-2	IGKV1-33	Human	407	98	0.06	N
VRC07-523LS	CD4bs	Y	IGVH1-2	IGKV1-33	Modified human	208	96	0.088	N
3BNC117	CD4bs	Y	IGVH1-2	IGKV1-33	Human	634	82	0.14	N
1-18	CD4bs	N	IGVH1-46	IGKV3-20	Human	119	97	0.048	Y
HJ16	CD4bs	N	IGVH3-10	IGKV4-1	Human	101	33	0.81	Y

^*a*^ N, no; Y, yes.

**Fig 4:**
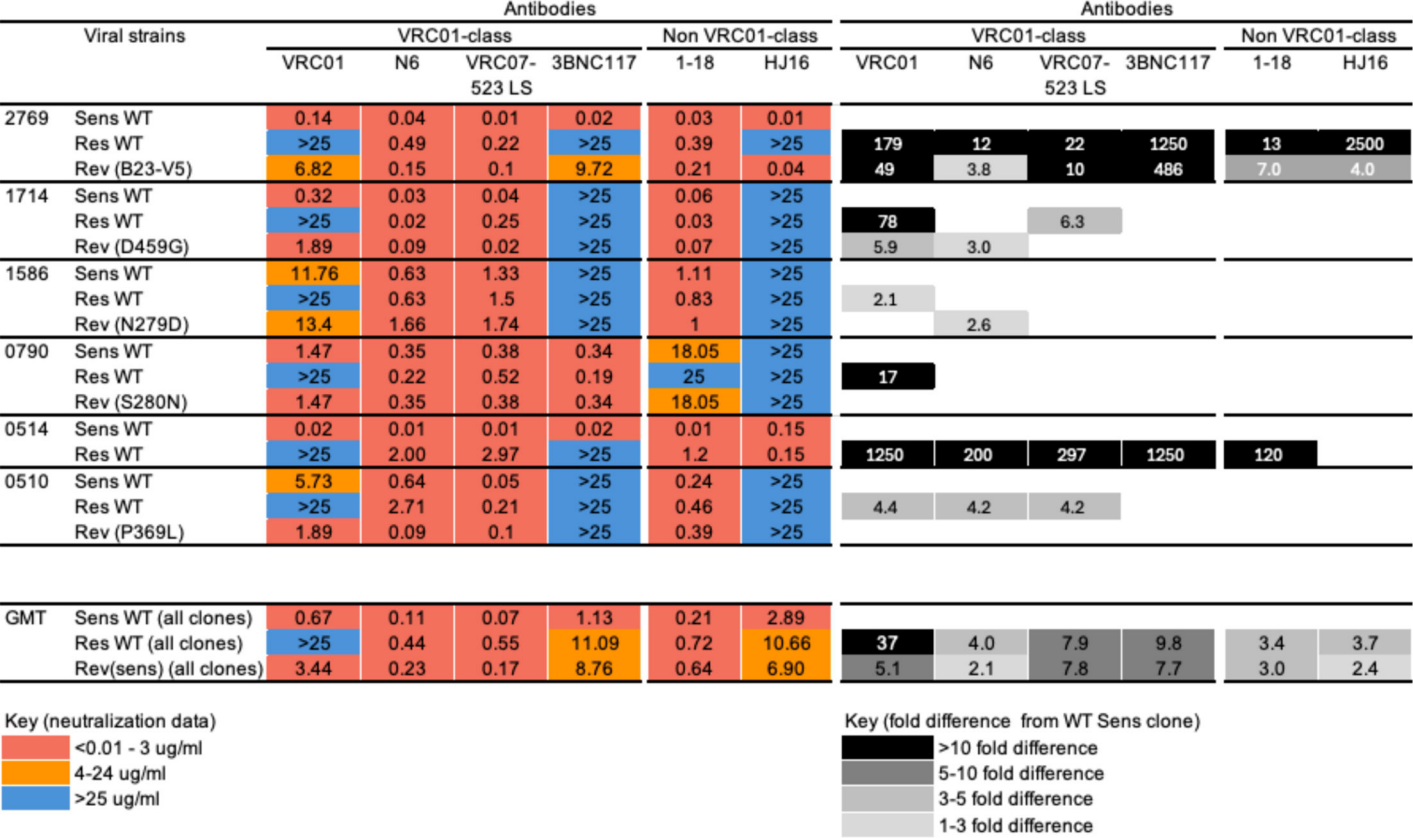
Inhibition profiles of VRC01-sensitive WT (Sens WT), VRC01-resistant WT (Res WT), and reverted mutants against diverse CD4-binding site bNAbs. Left panel, neutralization titers of the viruses against the CD4-binding site bNAb panel were measured as IC_50_ values. Sensitivity is represented in red for high (<0.01–3 ug/mL), intermediate in orange (4–24 ug/mL), and resistant in blue (>25 ug/mL). Right panel, fold loss in neutralization of the Res WT and Rev^Sens^ clones, compared to the Sens WT parental clones. The bottom panel (left) indicates the geometric mean titer for all Sens WT, Res WT, and Rev^Sens^ clones combined, and (right) fold loss in overall neutralization of the Res WT and Rev^Sens^ clones compared to the Sens WT parental clones.

As expected, given the increased potency and breadth of N6 and VRC07-523 LS compared to VRC01 (Table 1), all Sens WT clones were also sensitive to these antibodies. Furthermore, all Res WT clones, despite being resistant to VRC01, retained sensitivity to N6 and VRC07-523 LS at an IC_50_ of <3 ug/mL (Fig. 4). Similarly, all Rev^Sens^ clones were also sensitive to N6 and VRC07-523 LS.

However, the sensitivity of Res WT clones was fourfold and eightfold lower to VRC01-class bNAbs (N6 and VRC07-523 LS) compared to the matched Sens WT clones (with the geometric titer for Res WT clones of 0.44 and 0.55 ug/mL for N6 and VRC07-523 LS, respectively, compared to 0.11 and 0.07 ug/mL for Sens WT clones). The differential sensitivity was participant-specific, and particularly marked for 2769 and 0514, where fold differences between Sens WT and Res WT clones ranged from 12- to 297-fold. Thus, VRC01 escape mutations can have an impact on the potency of both N6 and VRC07-523 LS. This is further supported by the observation that the Rev^Sens^ clones were generally more sensitive to N6 and VRC07-523 LS compared to the Res WT clones, hence the reverted VRC01 resistance-associated mutations also impact recognition by next-generation VRC01-class bNAbs.

VRC01 Res WT clones also showed high-level sensitivity to non-VRC01-class bNAb, 1-18, which has high potency and breadth (Table 1), with titers ranging from 0.03 ug/mL to 1.2 ug/mL. An exception for 1-18 was participant 0790 where weak neutralization was observed for 0790 Sens WT (18 ug/mL) and the matched Rev^Sens^ pseudoviruses but complete resistance for the 0790 Res WT. We also observed reduced potency (13–120-fold) among Res WT clones from 2769 and 0514 compared to Sens WT clones, although with a lower fold change compared to VRC01-class bNAbs. The 2769 Rev^Sens^ was slightly more sensitive to 1-18 than the matched Res WT clone.

For the bNAb 3BNC117, which is reported to have 82% breadth (Table 1), neutralization was only observed for participants 2769, 0790, and 0514. For 0790, both clones and the Rev^Sens^ were sensitive to 3BNC117 (with less than a twofold difference in titer between Sens WT and Res WT). In contrast, for 2769 and 0514, only the Sens WT, but neither of the matched VRC01 Res WT clones was neutralized by 3BNC117, with fold loss in sensitivity >1,250 in both cases. This suggests that mutations associated with VRC01 resistance can mediate cross-resistance for 3BNC117.

The overall low breadth and potency of HJ16 (Table 1) is reflected in its general lack of neutralization against viruses from all six donors, with neutralization observed only for 2769 Sens WT and the Rev^Sens^ containing the entire β23-V5 region (but not the Res WT), and for both clones from 0514 (Sens WT and Res WT), where VRC01 resistance mutations had not been identified. In participant 2769, transfer of the β23-V5 region impacted all CD4-binding site bNAbs, with Rev^Sens^ also acquiring sensitivity to 3BNC117 and HJ16.

Overall, while VRC01-resistant clones retain sensitivity to several CD4bs bNAbs of clinical relevance, potency against the Res WT clones was reduced in a participant-specific manner. Fold changes between the Sens WT and Res WT were particularly high in 2769 and 0514 for all CD4bs mAbs. Of note, in the case of 2769, the transfer of sensitivity required the transfer of multiple mutations. In the case of 0514, so many mutations distinguished the two discordant envelopes that we could not define VRC01-resistant mutations, suggesting the possibility that multiple mutations may together mediate greater resistance to CD4bs antibodies. Overall, the reversion of mutations associated with VRC01 resistance partly or largely restored the potency of CD4bs-directed bNAbs, indicating that the mutations identified in this study confer some degree of cross-resistance to other bAbs.

## DISCUSSION

The HVTN703/HPTN081 AMP trial highlighted the important interplay between viral sensitivity and bNAb concentrations needed to block HIV-1 acquisition (4). From this single trial, we identified six women who had acquired multiple viral lineages that differed in their sensitivity to VRC01. By comparing the Env sequences of these resistant and sensitive viruses, we mapped features responsible for VRC01 neutralization resistance in five of these individuals. We found no common mechanisms of escape highlighting the diverse pathways by which HIV-1 can evade VRC01 neutralization. Although all VRC01-resistant clones and reverted mutants retained sensitivity to several other CD4bs bNAbs, they were frequently neutralized less potently than the matched VRC01-sensitive parental clones, indicating that mutations associated with VRC01 resistance confer some degree of cross-resistance.

The VRC01 resistance mutations identified were located in the VRC01-binding footprint including loop D, the CD4-binding loop, and the β23 and V5 loops. There are subtype-specific differences in VRC01 sensitivity with the subtype C viruses being more resistant overall than subtype B viruses (24). However, the mutations identified in this subtype C cohort were similar to those reported in other subtypes. As the mutations identified did not always completely restore sensitivity (or in one donor, required transfer of multiple mutations), other structural features of Env likely contributed to VRC01 resistance, and future studies introducing different combinations of mutations may identify alternate pathways to resistance.

Our study revealed that all VRC01-resistant clones retained sensitivity to other clinically relevant CD4-binding site bNAbs, such as VRC07-523 LS. N6 and 1-18. However, resistance profiles varied against other CD4-binding site bNAbs 3BNC117 and HJ16. Although N6 is a VRC01-class antibody, it overcomes VRC01 resistance mutations as it recognizes a slightly different epitope (33). Similarly, while both VRC01 and VRC07 were cloned from the same individual living with HIV, VRC07 was engineered to include mutations that enhanced its breadth, potency, and half-life compared to the parental mAb (34). Therefore, although N6, VRC07-523 LS, and 1-18 all target the CD4-binding site, they are more potent and broader than VRC01, targeting the virus at slightly different angles (17). These differences in the mode of binding and an increased affinity may explain why such antibodies are overall less affected by mutations that confer VRC01 resistance in these viruses. We noted, however, that although sensitivity to VRC07-523 LS and N6 was retained against VRC01-resistant envelopes, potency was lower in this study for all bNAbs. These data suggest that currently available bNAbs will likely provide adequate coverage in passive immunization studies. However, as VRC01 resistance is increasing at a population level (with contemporaneous viruses showing lower sensitivity compared to viruses from earlier in the pandemic), expanding this analysis to larger panels of viruses will be valuable, and monitoring this over time will be important (24).

Simultaneous circulation of VRC01-resistant and sensitive clones within a single participant has previously been described (35). The resistant clones studied here were from genetically linked but different infecting lineages. It is therefore likely that this resistant phenotype originated in the donor and did not evolve post-acquisition under VRC01 selective pressure, with the possible exception of one participant, where there was a close genetic distance (0.3%) between the lineages. These clones were derived from the first HIV-1 RNA-positive visit, when there was still VRC01 present (36). As VRC01 resistance mutations have been associated with a fitness cost in previous studies, future studies will need to assess the persistence of such mutations following breakthrough acquisitions in the context of waning bNAb levels (and whether they indeed incur a fitness cost) (14, 15).

Understanding the mutations that confer resistance to VRC01 and other bNAbs is crucial for the design of effective HIV-1 vaccines and therapies. The ability to predict and counteract resistance mutations can enhance the efficacy of bNAbs in both preventive and therapeutic settings. The overall retention of sensitivity to next-generation CD4-binding site bNAbs in VRC01-resistant viruses suggests that these newer antibodies could provide robust protection against HIV-1. Overall, this study provides insights into the mutations that distinguish HIV-1 Envs with discordant VRC01 phenotypes and their implications for cross-resistance to other bNAbs. The findings emphasize the need for a multifaceted approach in HIV-1 prevention, utilizing a combination of bNAbs to overcome resistance and achieve comprehensive protection. Further studies should investigate additional mutations and their impact on bNAb sensitivity, providing a more comprehensive understanding of resistance mechanisms.

## Data Availability

Sequencing data of HIV envelopes have been deposited in the GenBank database under accession numbers ON890964-ON890971, ON890991, ON890993, ON891051-ON891052, ON891042-ON891043, and ON891086-ON891087.

## SUPPLEMENTAL MATERIAL

Supplemental material jvi.01730-24-s0001.pdf Figures S1 and S2.

ASM does not own the copyrights to Supplemental Material that may be linked to, or accessed through, an article. The authors have granted ASM a non-exclusive, world-wide license to publish the Supplemental Material files. Please contact the corresponding author directly for reuse.
