# Supplementary material for: Resistance mutations that distinguish HIV-1 envelopes with discordant VRC01 phenotypes from multi-lineage infections in the HVTN703/HPTN081 trial: implications for cross-resistance
Source: J Virol. 2025 Jan 16;99(2):e01730-24. doi: 10.1128/jvi.01730-24 (PMC11852940; doi:10.1128/jvi.01730-24)
Supplement: Supplemental material — Figures S1 and S2. [file jvi.01730-24-s0001.pdf]

Supplementary Figure 1

Cloned plasmid name	VRC01 IC_{50} (ug/ml)	VRC01 IC_{80} (ug/ml)
**V703_2769_050_RE_p01s**	**11.76**	**>25**
**V703_2769_050_RE_pblib002_s**	**0.12**	**0.41**
**V703_1714_080_RE_pblib002_s**	**>25**	**>25**
**V703_1714_080c**	**0.29**	**1.03**
**V703_1586_210_RE_sga3E8_s**	**>25**	**>25**
**V703_1586_210_RE_pblib002_s**	**8.35**	**>25**
**V703_0790_190_RE_pblib002_s**	**22.63**	**>25**
**V703_0790_190_RE_e5G1s**	**4.39**	**13.02**
**V703_0514_150Es_F4_V03_024**	**>25**	**>25**
V703_0514_150_RE_pblib004_s	>25	>25
V703_0514_150s_6C1	>25	>25
**V703_0514_150Es_A4_V02_028**	**0.04**	**0.14**
**V703_0510_260_RE_pblib003_s**	**>25**	**>25**
**V703_0510_260_RE_pblib002_s**	**4.55**	**15.93**
V703_0510_260_RE_pblib001_s	3.68	13.82

Supplementary Figure 1. Neutralization sensitivity of all clones within the phylogenetic clusters, with clones selected for further study shown in bold. IC_{50} and IC_{80} values measured in ug/ml.

Supplementary Figure 2 A-F. Amino acid alignments of clones forming the basis of these studies, with mutated sites denoted (panels on next page)

Supplementary Figure 2A

```
V703_0790_pblib002 Resistant WT  MRVMGIWRNCPPWWMWGILGFWMLMICNGAGNLWVTVYYGVPVWKEAKTTLFCASDAKAYVKEAHNIWATHACVPTDPNPQEIVLKNVTENFNMWENNMV
H703_0790_e5G Sensitive WT       ....................................................................................................

V703_0790_pblib002 Resistant WT  NQMHEDIINLWEQSLKPCVKLTPLCVTLNCSNANYSSDARYNGSSIEIKNCTFNASTEIKDKKYKQRALFYALDLVQINSSTYRLVHCNSSTIAQACPKI
H703_0790_e5G Sensitive WT       ....................................................................................................

                                                                                        280
V703_0790_pblib002 Resistant WT  SFDPIPIHYCAPAGYAILKCNDKTFNGTGPCHNVSTVQCTHGIKPVVSTQLLLNGSLAEKEIVIRSKNLTDSTKTIIVHFNKSVEITCTRPNNNTRKSIR
H703_0790_e5G Sensitive WT       .......................................................................N............................
                                                                                     Loop D

V703_0790_pblib002 Resistant WT  IGPGQTFFAAGDIIGNIREAHCNISKEAWNRTLQRVGEELEKHFPNKTITFNSSSGGDLEITTHMFNCGGEFFYCNTSGLFNGTYNSTYNSTELHNATIT
H703_0790_e5G Sensitive WT       ....................................................................................................
                                                                   CD4 binding loop

V703_0790_pblib002 Resistant WT  IPCRIRQIINMWQEVGRAMYAPPIAGNITCKSNITGLLLVRDGGRDQDKNSSIETFRPQGGNMKDNWRSELYKYKVVEIKPLGVAPTKAKRRVVEREKRA
H703_0790_e5G Sensitive WT       ....................................................................................................
                                          CD4 contact                      B23 and V5 loop

V703_0790_pblib002 Resistant WT  AGIGALLIGFLGAAGSTMGAASITLTVQARQLLSGIVQQQNNLLRAIEAQQHLLQLTVWGIKQLQARVLAVERYLSDQQLLGLWGCSGKLICTTAVPWNF
H703_0790_e5G Sensitive WT       ....................................................................................................

V703_0790_pblib002 Resistant WT  SWSNRSQDKIWKNMTWMQWEREIDNYTGTIYKLLEESQFQQETNEKDLLALDSWQNLWSWFDITKWLWYIKIFIMIVGGLIGLRIIFAVLSIVKRVRQGY
H703_0790_e5G Sensitive WT       ....................................................................................................

V703_0790_pblib002 Resistant WT  SPLSFQTLTPNPREIDRLGRIEEEGGEQDRARSIRLVNGFLPLVWDDLRNLCLFSYHRLRDCILVTARVVELLGRSSLRGLQRGWEALKYLGSLVQYWGL
H703_0790_e5G Sensitive WT       ....................................................................................................

V703_0790_pblib002 Resistant WT  ELKKSAISLFDSLAITVAEGTDRIIEIIQRLCRAIRNIPRRIRQGFEAALL
H703_0790_e5G Sensitive WT       ...................................................
```

Supplementary Figure 2B

```
V703_1586_ga3E8 Resistant WT      MRVMGTMRNWQHWGIWGILGFWMLMICNVVGDLWVTVYYGVPVWKDAKTTLFCASDAKAYEKEVHNVWATHACVPTDPNPQEIVLANVTENFNMWKNDMV
V703_1586_pblib002 Sensitive WT   .....................................................................................G..............

V703_1586_ga3E8 Resistant WT      DQMHEDIISLWDESLKPCVKLTPLCVTLNCTNATASSNGNGTSGTIEDSMQGKIKNCSFNATTEIRDKKLKVSALFYRVDIVPLNGIEDNSTNYTEYRLI
V703_1586_pblib002 Sensitive WT   ....................................................................................................

                                                                                                      279
V703_1586_ga3E8 Resistant WT      NCNTSTLTQACPKVTFEPIPIHYCAPAGFAILKCNNETFNGTGPCQNVSTVQCTHGIRPVVSTQLLLNGSLAEGEIMIRSENLTNNAKTIIVHFNKSVDI
V703_1586_pblib002 Sensitive WT   ....................................................................................D...............
                                                                                                       Loop D

V703_1586_ga3E8 Resistant WT      VCTRPNNNTRKSVRIGPGQVFYTNDIIGDIRQAHCNITRQNWTDTLQKVAEKLREKFPNKTIIFNSSSGGDLEIVTHSFNCGGEFFYCNTSGLFNRTFYA
V703_1586_pblib002 Sensitive WT   .......................................K....................K......................................E
                                                                                         CD4 binding loop

V703_1586_ga3E8 Resistant WT      NETS----SNDTLGQNENITLPCRIKQIINMWQEVGRAMYANPIAGNITCKSNITGLLLVRDGGINGTNDTETFRPQGGNMKDNWRSELYKYKVVEIKPL
V703_1586_pblib002 Sensitive WT   .G..SNDT............................................................................................
                                                            CD4 contact                B23 and V5 loop

V703_1586_ga3E8 Resistant WT      GIAPTKAKRRVVEREKRAVGIGAVFLGFLGVAGSTMGAASLTLTVQARQLLSGIVQQQSNLLRAIEAQQHMLQLTVWGIKQLQARVLAIERYLKDQQLLG
V703_1586_pblib002 Sensitive WT   ....................................................................................................

V703_1586_ga3E8 Resistant WT      LWGCSNKLICTTTVPWNSSWSNKTYNYIWNNMTWMQWDAEIDNYTNTIYRLLEVSQNQQEQNEKDLLELDKWNNLWSWFKITNWLWYIKIFIMIVGGLIG
V703_1586_pblib002 Sensitive WT   .........................E...G......................................................................

V703_1586_ga3E8 Resistant WT      LRIVLSVLSIVNRVRQGYSPLSFQTLPPNQRGLDRLGGIEEEGGEQDRSRSIRLVSGFLPIAWDDLRNLCLFLYHRLRDCTLIAARALELLGRSSLKGLQ
V703_1586_pblib002 Sensitive WT   .......C........................................N..................S..I........FI...................

V703_1586_ga3E8 Resistant WT      RGWEILKYLGGLAQYWGLELKKSAISLLDTIAIAVAEGTDRIIEFLRGICRAIRNLPRRIRQGFEASLL
V703_1586_pblib002 Sensitive WT   .....................................................................
```

Supplementary Figure 2C

```
V703_1714_pblib002 Resistant WT    MRVRGILRNYQQWWIWGVLAFWMLIIGNVGENLWVTVYYGVPVWREAKTTLFCASDAKAYEREVHNVWATHACVPTDPSPQEIVLENVTENFNMWKNDMV
H703_1714_080s Sensitive WT        ....................................................................................................

V703_1714_pblib002 Resistant WT    DQMHEDIISLWDQSLKPCVKLTPLCVTLTCKNVTNTIKDG-ETIKNCSFNVTTERRDKRKGEYALFYTHDLVPLNENGNSNSSEYILISCNTSVIKQACP
H703_1714_080s Sensitive WT        .................................N...G..A...........................Q...............................

V703_1714_pblib002 Resistant WT    KVSFDPIPIHYCAPAGYAILKCNDNKFNGTGLCNNVSTVQCTHGIKPVVSTQLLLNGSLAKEEIIIRSKNLTDNTKTIIVQFNESVEIVCIRPNNNTRKS
H703_1714_080s Sensitive WT        ........................................................................................E...........
                                                                                                        Loop D

V703_1714_pblib002 Resistant WT    IRIGPGQVFYANDIIGDIRKAYCNISISKWNKTLTEVGKKLREHFSNKTIRFTSPSPGGDPEITLHSFNCGGEFFYCNTSKLFTSKLFPNGTYLFNNDTE
H703_1714_080s Sensitive WT        ...........T.............N....DR.................................................------------....N..
                                                                                         CD4 binding loop

                                                                                     459  462
V703_1714_pblib002 Resistant WT    DNGTITINCKIKQIINMWQRVGLTMYAPPIQGNITCTSNITGLLLERDGDANESNTETFRPTGGDMRDNWRSELYKYKVVEIKPLGIAPTAAKRRVVERE
H703_1714_080s Sensitive WT        .................................................G..D...............................................
                                                 CD4 contact                 B23 and V5 loop

V703_1714_pblib002 Resistant WT    KRAVGIGAVLLGFLGAAGSTMGAASITLTVQARQLLSGIVQQQNNLLRAIEAQQHMLQLTVWGIKQLQARVLAIERYLQDQQLLGIWGCSGKLICTTNVP
H703_1714_080s Sensitive WT        .........................................................................V.......................D..

V703_1714_pblib002 Resistant WT    WNRSWSNKTQEEIWGNMTWMQWDKEINNYTGMIYKLLEESQNQQDINEKDLLALDNWKNLWNWFDITNWLWYIKIFIMIVGGLIGLRIIFAVLSIVNRVR
H703_1714_080s Sensitive WT        ..................................T......I...K......................................................

V703_1714_pblib002 Resistant WT    QGYSPLSFQTLIPNPRGPDRLGRIEEEGGEQDRDRSIRLVSGFLALAWDDLRSLCLFSYHRLRDFILVTARAVELLGRSSLKGLQKGWQALKYLGSLVQY
H703_1714_080s Sensitive WT        ....................................................................................................

V703_1714_pblib002 Resistant WT    WGLELKKSAISLLDAIAIAVAEGTDRILELLQRLCRAIWNIPRRIRQGFEAALQ
H703_1714_080s Sensitive WT        ......................................................
```

Supplementary Figure 2D

```
H703_2769_p01s Resistant WT       MRVRGMLRNCPPWWIWSILGLWMVIMGNEEKDLWVTVYYGVPVWREAKTTLFCASDAKSYEKEAHNIWATHACVPTDPNPQEVFLDNVTENFNMWKNDMV
V703_2769_pblib002 Sensitive WT   ...................................................................................V.K..............

H703_2769_p01s Resistant WT       DQMHEDIISLWDESLKPCVKLTPLCVTLNCSDVTVSQNETT--KYSTINANQTEQMKNCSFNTTTVIRDKKKQEYALFYKLDIEALPKNKNSNNSDSNSN
V703_2769_pblib002 Sensitive WT   ...............................K.N.NDTQIAEIRKGIV.TS...E.......A.A.LKN...K...........P.H..G...D.-----

H703_2769_p01s Resistant WT       DSNEYILINCNTSTIAQACPKITFEPIPIHYCAPAGYAILKCNNNTFNGTGPCNNVSTVQCTHGIKPVVSTQLLLNGSLVEGKDIIIRSENITDNVKTII
V703_2769_pblib002 Sensitive WT   ---K.........A.............................DKE.................................A.-DG.........N......
                                                                                                                      Loop D

H703_2769_p01s Resistant WT       VHLNESVEINCTRPGNNTRKSVRIGPGQTFYATGAIIGDIRQAHCNISRAKWNITLERVKTKLGEYFSN-STIVFKPPVPGGDPEITTHSFNCGGEFFYC
V703_2769_pblib002 Sensitive WT   ....K..G.............I......A.....D.............ETE..A......E...Q....-K.TISFE.AS.....VI.............
                                                                                                      CD4 binding loop
                                                                        432
H703_2769_p01s Resistant WT       NTTDLFSANATTNSSATGTITLQCRIKQIINMWQQVGRAIYAPPIAGNITCNSTITGLLLTRDGGETNKTNNSTEEIFRPGGGDMRDNWRSELYKYKVVE
V703_2769_pblib002 Sensitive WT   ...K...---S.H.AVN.....P.K............Q...............N...........---NV....-.T.......................
                                                              CD4 contact                    B23 and V5 loop

H703_2769_p01s Resistant WT       IKPLGVAPTRAKRRVVEREKRAVGIGAMFLGFLAAAGSTMGAASITLTVQARQLLSGIVQQQSNLLRAIEAQQHLLQLTVWGIKQLQTRVLALERYLKDQ
V703_2769_pblib002 Sensitive WT   .........G.R............L..V.I...G................................................V..............T..

H703_2769_p01s Resistant WT       QLLGIWGCSGKLICTTSVPWNNSWSNKSLGDIWENMTWMQWDREVSNYTNTIYMLLEKSQIQQEQNEKDLLSLNSWGSLWNWFSITQWLWYIKIFIMIVG
V703_2769_pblib002 Sensitive WT   ................N................D...................R...E..N.........................R.............

H703_2769_p01s Resistant WT       GLIGLRIIVAVISTVNRVRQGYSPLSFQTLYPSPGGPDRLGRIEEEGGEQDKNRSVRLVNGFLPLVWDDLRSLCLFCYHRLRDCILIATRAVEILGRSSL
V703_2769_pblib002 Sensitive WT   ..............................I......................K.........A...................F................

H703_2769_p01s Resistant WT       RGLQRGWETLKYLGNLVLYWVLELKKSAISLLDTLAITVAEGTDRIIELGQRICRAIISIPRRIRQGFEAALQ
V703_2769_pblib002 Sensitive WT   K.............S.....G..........I.....A..............T....RN..............
```

Supplementary Figure 2E

```
V703_0510_pblib003 Resistant WT  MRVRGILKNYQQWWIWGILGFWMLMIYNVGGNLWVTVYYGVPVWKDAKTTLFCASDAKAYEKEVHNVWATHACVPTDPNPQEIVLGNVTENFNMWKNDMV
V703_0510_pblib002 Sensitive WT  ....................................................................................................
V703_0510_pblib001 Sensitive WT  ...K................................................................................................

V703_0510_pblib003 Resistant WT  DQMHEDIISLWDQSLKPCIKLTPLCVTLNCSNANSTSVNSTYNSNKNGEIQNCSFNATTEIHDRKKKEYALFYKLDIVPLEGSNNTT-YRLINCNSSTVT
V703_0510_pblib002 Sensitive WT  ..................R............S..R.I.DPS..---R..............K.K.R..........V........N.Y............
V703_0510_pblib001 Sensitive WT  .....................................................................................-............

V703_0510_pblib003 Resistant WT  QACPKISFDPIPIHYCAPAGYAILKCNNQTFNGTGPCQNVSTVQCTHGIKPVVSTQLLLNGSLAEGGETRIRSEDLTNNAKIIIVHLNESVKITCVRPNN
V703_0510_pblib002 Sensitive WT  .....V...........................................................................T.N...I....
V703_0510_pblib001 Sensitive WT  ..........................................................................I.................T.N...I....
                                                                                                       Loop D
                                                                                               369 371
V703_0510_pblib003 Resistant WT  NTRKSIRIGPGQAFYATNDIIGDIRQAYCNISKANWNKTLKWVGQKLKEHFSNKTIEFRPSSGGDPEITTHSFNCRGEFFYCNTSQLFNSTYNSSSTYND
V703_0510_pblib002 Sensitive WT  ......................N....H.....EE.DN..QK.R...G........K.Q......L.V..............D..R.....C.N...C..
V703_0510_pblib001 Sensitive WT  ......................N....H.....EE.DN..QK.R...G........K.Q......L.V..............D..R.....C.N...C..
                                                                                         CD4 binding loop

V703_0510_pblib003 Resistant WT  TKGDKN--ITLPCRIKQIINMWQEVGKAMYAPPIEGELKCVSNITGLLLVRDGGNETNGTGTEIFRPGGGNMKDNWRSELYKYKVVEIKPLGIAPTEAKR
V703_0510_pblib002 Sensitive WT  ..E.G.NI..I.........................................................................................
V703_0510_pblib001 Sensitive WT  ..E.G.NI............................................................................................
                                        CD4 contact                 B23 and V5 loop

V703_0510_pblib003 Resistant WT  RVVEREKRAVGIGAVFLGFLGAAGSTMGAASITLTVQARQLLSGIVQQQSNLLRAIEAQQHLLQLTVWGIKQLQTRVLSIERYLKDQQLLGIWGCSGKLI
V703_0510_pblib002 Sensitive WT  ....................................................................................................
V703_0510_pblib001 Sensitive WT  ....................................................................................................

V703_0510_pblib003 Resistant WT  CTTNVPWNSSWSNKSYQEIWDNMTWMQWDREIANYTQTIYGLLEDSQIQQEQNEKDLLALDKWQNLWSWFSITQWLWYIKIFIMIVGGLIGLRIIFAVLS
V703_0510_pblib002 Sensitive WT  .........................................................................H..........................
V703_0510_pblib001 Sensitive WT  ................T........................................................H..........................

V703_0510_pblib003 Resistant WT  IVNRVRQGYSPLSFQTLTPNPRELDRLERIEEGGGEQDRDRSIRLVNGFLPLVWDDIRNLCRFSYHQLRDFILVTARVVELLGRSSLRGLQRGWEVLKYL
V703_0510_pblib002 Sensitive WT  ...........................G..................S.....................................................
V703_0510_pblib001 Sensitive WT  ....................................................................................................

V703_0510_pblib003 Resistant WT  GGLVQYWGQELKKSAISLLDTTAIAVAEGTDRIIEFIQRICRAIRNIPTRIRQGFEAALL
V703_0510_pblib002 Sensitive WT  .S..........................................................
V703_0510_pblib001 Sensitive WT  ............................................................
```

Supplementary Figure 2F

```
H703_0514_F4_V03 Resistant WT      MRVKGTLRNWQQWWIWGILGFWMVLICNVGGNLWVTVYYGVPVWKEAKTTLFCASDAKAYDKEVHNVWATHACVPTDPNPQEIELKNVTENFNMWKNDMV
V703_0514_pblib004 Resistant WT    .......................LM...................R......................................K.E..............
H703_0514_6C1 Resistant WT         .......................LM...................R......................................K.E..............
H703_0514_A4_V02 Sensitive WT      .......................LM.Y.................................E.....I................V.E..............

H703_0514_F4_V03 Resistant WT      EQMHEDIISLWDQSLKPCVKMTPLCVTLNCTNAIS-------TKTNSTSTVNSTSSPNVNISTSTTDEMKNCSFNVTTELRDKSKKEYALFYRLDVVPL-
V703_0514_pblib004 Resistant WT    D................................RANITSTNG.N.....SS..........--................................IL..E
H703_0514_6C1 Resistant WT         D................................RANITSTNG.N.....SS..........--................................IL..E
H703_0514_A4_V02 Sensitive WT      D...................L.........A.----------IS..T..A-------KI.N..NNNN........I.......R....V......I...-
                                                                                                                                 281
H703_0514_F4_V03 Resistant WT      GNDTGNFSDYRLINCNSSTITQACPKVTFDPIPIHYCAPAGYAILKCNNKTFNGTGPCNNVSTVQCTHGIKPVVSTQLLLNGSLAEGEIIIRSKNLTDNT
V703_0514_pblib004 Resistant WT    K.E....N.....................................................................................E.....G
H703_0514_6C1 Resistant WT         K.E....N.....................................................................................E.....G
H703_0514_A4_V02 Sensitive WT      ..E--.S.E.......T.A........S........................K......K..........................E..........E.S
                                                                                                                        Loop D

H703_0514_F4_V03 Resistant WT      KIIIVHLNQTVEIKCTRPQNNTRKSVRIGPGQTFYAPGDIIGDIRQAHCNISRSDWQQAMKNVSKKLSEKFRELFNTTTLVFKPPVGGDPEIVTHSFNCQ
V703_0514_pblib004 Resistant WT    .T..A...R..K.M............................N.......V.KV...W.IQ..----...L...Y....I..ES...............R
H703_0514_6C1 Resistant WT         .T..A...R..K.M............................N.......V.KV...W.IQ..----...L...Y....I..ES...............R
H703_0514_A4_V02 Sensitive WT      ........K..K.V.......................T.EV......Y...N..E.E.TLH..----...LK.H..-..II.E.SA...L.V.......R
                                                                                                                  CD4 binding loop
                                                                                  429
H703_0514_F4_V03 Resistant WT      GEFFYCNTSGLFNGIYSNGTYNDT-YNDTETTITLPCRIKQIINMWQGVGRAMYASPIAGNITCTSNITGLLLTRDGGINNTNTNETFRPGGGDMRDNWR
V703_0514_pblib004 Resistant WT    ..............T.-.....EIDN..........................................................................
H703_0514_6C1 Resistant WT         ..............T.-.....EIDN..........................................................................
H703_0514_A4_V02 Sensitive WT      ..............T.-.....NS-NP.----.I...K.........E.......P........V.............QGTNS.S...............
                                                                               CD4 contact                    B23 and V5 loop

H703_0514_F4_V03 Resistant WT      SELYKYKVVEIKPLGIAPNMAKRRVVEREKRAVGIGAVLLGFLGAAGSTMGAASIALTVQARQLLSGIVQQQNNLLRAIEAQQHMLQLTVWGIKQLQARV
V703_0514_pblib004 Resistant WT    ...................T..................................V.............................................
H703_0514_6C1 Resistant WT         ...................T..................................V.............................................
H703_0514_A4_V02 Sensitive WT      ...............V..TT............A....M.F............................................................

H703_0514_F4_V03 Resistant WT      LAIERYLKDQQLLGLWGCSGKLICTTTVPWNSSWSNKSIEDIWGNMTWMQWDREIDNYTSLIYSLLEESQRQQEKNEQDLLALDSWNSLWNWFDISKWLW
V703_0514_pblib004 Resistant WT    ..........................A.........RTKD...N.......E...N...NI......K..S.............................
H703_0514_6C1 Resistant WT         ..........................A.........RTKD...N.......E.......NI......K..S.............................
H703_0514_A4_V02 Sensitive WT      ..........................A...........GT...D...........N....I.........S......K......................

H703_0514_F4_V03 Resistant WT      YIKIFIMIVGGLIGLRIIFAVLSIVNRVRQGYSPLSFQTLIPNPRGPDRPRGIEEEGGEQDRDRSIRLVNGFLALAWDDLRSLCLFSYHRLRDFILVTAR
V703_0514_pblib004 Resistant WT    ......................................................................S..........................A..
H703_0514_6C1 Resistant WT         ......................................................................S..........................A..
H703_0514_A4_V02 Sensitive WT      .........................................T........LER............................................AV.

H703_0514_F4_V03 Resistant WT      AVELLGRSSLKGLQRVWEVLKYLGNLVQYWGLELKKSAISLLDTIAITVAEGTDRIIEIIQRFFRAIFNIPRRIRQGFEAALL
V703_0514_pblib004 Resistant WT    ...................................................................................
H703_0514_6C1 Resistant WT         ...................................................................................
H703_0514_A4_V02 Sensitive WT      .......................R...........RN..........................C.V.Y...............
```
